# The combination of MELD score and ICG liver testing predicts length of stay in the ICU and hospital mortality in liver transplant recipients

**DOI:** 10.1186/1471-2253-14-103

**Published:** 2014-11-15

**Authors:** Stephanie Klinzing, Giovanna Brandi, Paul A Stehberger, Dimitri A Raptis, Markus Béchir

**Affiliations:** Surgical Intensive Care Medicine, University Hospital of Zurich, Raemistrasse 100, CH-8091 Zurich, Switzerland; Department of Visceral- and Transplantation Surgery, University Hospital of Zurich, Zurich, Switzerland

**Keywords:** MELD score, Indocyanine green liver testing, Hospital mortality, Length of stay in the ICU

## Abstract

**Background:**

Early prediction of outcome would be useful for an optimal intensive care management of liver transplant recipients. Indocyanine green clearance can be measured non-invasively by pulse spectrophometry and is closely related to liver function.

**Methods:**

This study was undertaken to assess the predictive value of a combination of the model of end stage liver disease (MELD) score and early indocyanine plasma disappearance rates (ICG-PDR) for length of stay in the intensive care unit (ICU), length of stay in the hospital and hospital mortality in liver transplant recipients.

**Results:**

Fifty consecutive liver transplant recipients were included in this post Hoc single-center study. ICG-PDR was determined within 6 hours after ICU admission. Endpoints were length of stay in the ICU, length of hospital stay and hospital mortality. The combination of a high MELD score (MELD >25) and a low ICG-PDR clearance (ICG-PDR < 20%/minute) predicts a significant longer stay in the ICU (p = 0.004), a significant longer stay in the hospital (p < 0.001) and a hospital mortality of 40% vs. 0% (p = 0.003).

**Conclusion:**

The combination of MELD scores and a singular ICG-PDR measurement in the early postoperative phase is an accurate predictor for outcome in liver transplant recipients. This easy-to-assess tool might be valuable for an optimal intensive care management of those patients.

## Background

In 2002, the United Network for Organ Sharing introduced a new allocation policy for cadaveric liver transplants based on the end-stage liver disease (MELD) scoring system [1].

However, while the MELD score is an accurate predictor of waiting list mortality [2, 3], recipient-derived methods including MELD [4–7] and, for example, the Child-Pugh score [5, 8] poorly predict mortality in liver transplant recipients. Similarly graft derived methods like the donor risk index, also has poor predictive value [9]. The 2008 Survival Outcomes Following Liver Transplant (SOFT) Score [10], on the other hand, incorporates 18 recipient- and donor- derived factors as well as operative factors and, as a result, accurately predicts short-term recipient survival following liver transplantation.

Due to the improvement in surgical techniques and a reduction in allograft rejection, the 1-year patient and graft survival is 90% [4, 11, 12], with the highest mortality rate during the early postoperative period in the critical care unit [13, 14]. Major complications occurring in the early postoperative phase are mainly due to graft non-function, acute rejection, sepsis, neurological complications and haemorrhagic shock.

Only limited date on the critical care management of and complications in liver transplant recipients are available [15–18]. Therefore, an easy-to-assess test with good accuracy would be valuable for outcome prediction and risk stratification in the postoperative phase of liver transplant patients.

High MELD scores reflect the severity of liver disease before transplantation and are associated with postoperative morbidity and complication [4, 19]. Less is known about factors which predict postoperative outcome of liver transplant recipients. The assessment of post-transplant liver function traditionally is based on several nonspecific liver function tests that are difficult to interpret and need serial observation [20]. Indocyanine green (ICG) is closely correlated with hepatic function due to its hepatic metabolization. It has proven to predict outcome in critically ill [21] and septic patients [22], but its relevance in liver transplantation is elusive [23, 24]. Classically ICG measurements were performed either by spectrophotometry [25] or with a fiber-optic aortic catheter placed through the femoral artery [26]. Both methods correlate well with graft function, but are time consuming, expensive or invasive. On the other hand, the method of non-invasive ICG measurement based on pulse dye measurement using a finger-clip correlates well with the classical measurements [27], but it is non-invasive and easy to use.

Therefore we evaluated the predictive power of preoperative MELD, postoperative ICG measurement and a combination of these values regarding length of stay in the ICU, length of stay in the hospital and mortality by performing single and combined ROC analysis.

## Methods

From September 2007 to June 2009, 50 consecutive patients who underwent transplantation at our center and received an ICG test within six hours after admission to the ICU were included in this Post Hoc analysis. Following approval by the local ethic committee (KEK Kantonale Ethik Kommission [Cantonal Ethical Committee] 4, Canton Zurich), all patients gave written informed consent before transplantation for postoperative data analysis.

### ICG liver test

The ICG liver testing was performed noninvasively by pulse spectrophotometry (LiMON®, Pulsion Medical Systems AG, Munich, Germany). After intravenous injection ICG-bolus (0.25 mg/kg; ICG Pulsion Medical Systems AG, Munich, Germany), plasma ICG concentrations were determined by pulse spectrophotometry with a finger-clip sensor that detects two near-infrared wavelengths.

The plasma disappearance rate of ICG (ICG-PDR) was calculated automatically by the time course of the blood ICG concentration (normal value: ICG-PDR ≥ 16%/minute).

After ICU admission, volume was assessed with passive leg raise test and focused transthoracic echocardiography and corrected if necessary. ICG-PDR measurements were performed within 6 hours from admission to the ICU.

### Operative technique

All livers were transplanted without a veno-venous bypass, as described by McCormack and colleagues [28].

### Baseline data

Gender, age and body mass index were collected for recipients and donors. For recipients, uncorrected MELD scores, SAPS II as well the incidence of renal replacement therapy (RRT) and hepato-renal-syndrome (HRS) before transplantation were recorded. The values of creatinine, haematocrit, platelets, INR and bilirubin immediately prior to transplantation were gathered. Creatinine values for patients on RRT were excluded. HRS was defined according to the definitions by Arroy [29] and Salerno [30].

The incidence of cadaveric or living donors as well as extended donor criteria was registered. Extended donor criteria (marginal grafts) were defined as age of 65 years or older, cold ischemia time of 720 minutes or longer, or biopsy-proven steatosis (micro- or macrovascular in ≥60% of hepatocytes or ≥30% macrovascular steatosis) [31].

### ICU data

The ICG-PDR was measured within the first 6 hours after admission, the factor V 24 hours after transplantation, and the peak values for bilirubin during the first postoperative week were recorded.

Data concerning length of ICU and hospital stay were collected and hospital mortality was recorded.

### Statistical analysis

Continuous variables were compared with the Mann-Whitney U, or Kruskal-Wallis tests, where appropriate. Differences among proportions were compared using the Fischer’s Exact or the Pearson χ^2^ tests, where appropriate. All p values were two-sided and considered statistically significant if p ≤0.05. Sensitivity, specificity, accuracy, diagnostic odds ratio (OR), and the receiver operator characteristic (ROC) curve were also calculated [32–36]. According to the results, a cut off of 4 days for length of stay in the ICU and 37 days for length of stay in the hospital was determined. Thereafter a logistic regression model including gender, age, BMI and the combined MELD/ICG score was performed. Statistical analysis was performed using SPSS Statistics version 20 (SPSS: An IBM Company, Chicago IL, 2012).

## Results

Demographic data and baseline characteristics are shown in Table 1. The postoperative ICU results are presented in Table 2.

**Table 1:** **Demographic data of the recipients and donors (n = 50)**

	Recipient	Donor
Male (%)	37 (74%)	36 (72%)
Female (%)	13 (26%)	14 (28%)
Age (yrs)	51.3 ± 11.1 (16 - 67)	53.2 ± 17.2 (19 - 86)
BMI (kg/m^2^)	25.7 ± 4.73 (16.6 - 42.9)	24.3 ± 3.3 (16.0- 31.0)
MELD	21 ± 10.4 (6 - 40)	
RRT before TPL (%)	8 (16%)	
HRS before TPL (%)	17 (34%)	
SAPS II	30 ± 19 (0 - 91)	
Creatinine (μmol/l)	121 ± 117 (40 - 814)	
Hematocrit (%)	31.4 ± 7.6 (18.8 - 49.6)	
Platelets (10^3^/μl)	106 ± 65 (33 - 324)	
INR	1.5 ± 0.6 (0.9 - 4.3)	
Bilirubin (μmol/l)	148 ± 198 (5 - 875)	
Etiology of liver disease		
HCV (%)	17 (34%)	
HBV (%)	3 (6%)	
HCC (%)	10 (20%)	
Alcoholic liver cirrhosis (%)	6 (12%)	
Cholangiocarcinoma (%)	2 (4%)	
Others^1^ (%)	12 (24%)	
Cadaveric Donor (%)		44 (88%)
Living Donor (%)		6 (12%)
Extended donor graft criteria (%)		16 (32%)

Data expressed as mean ± standard deviation (range). Abbreviations: BMI, body mass index; RRT, renal replacement therapy; TPL, transplantation; HRS, hepato-renal syndrome; SAPS, simplified acute physiology score; INR, international normalized ratio; HCV, hepatitis C virus; HBV, hepatitis B virus; HCC, hepatocellular carcinoma. Footnote: ^1^) encompasses primary biliary cirrhosis, primary sclerosing cholangitis, autoimmune hepatitis liver cirrhosis, Morbus Wilson, alpha-1-antitrypsin-defiency, acute liver failure, cryptogenic liver cirrhosis, Morbus Osler, polycyclic liver disease, recurrent intrahepatic cholestasis, vanishing bile duct syndrome, haemangioendothelioma.

**Table 2:** **Postoperative ICU data (n = 50)**

ICG-PDR (%/min)^1^	19.5 ± 7.7 (4.2 - 34)
Bilirubin (μmol/l)^2^	133 ± 115 (17 - 568)
Factor V (%)^3^	57 ± 29 (4 - 114)
ICU stay (days)	11.6 ± 21.9 (1 - 93)
Hospital stay (days)	31.4 ± 28.0 (8 - 128)
Hospital Mortality (%)	4 (8%)

Data expressed as mean ± standard deviation (range). Abbreviations: ICG-PDR, indocyanine green plasma disappearance rate. Footnotes: ^1^) determined within 6 hours after ICU admission, ^2^) peak values within 7 days, ^3^) after 24 hours.

Twenty-seven patients stayed in the ICU for more than four days and thirteen patients had a hospital stay of more than thirty-seven days (Table 3). Patients with an ICU stay longer than four days were characterized by a significantly higher MELD score (p = 0.007), significantly decreased ICG-PDR (p = 0.001), significantly elevated peak bilirubin within the first postoperative week (p = 0.03) and significantly decreased factor V within the first 24 hours after operation (p = 0.03). Patients with a prolonged hospital stay over 37 days were characterized by significantly higher MELD score (p < 0.001) and significantly elevated peak bilirubin within the first postoperative week (p = 0.008).

**Table 3:** **ICU and hospital stay grouped data**

	ICU stay ≤4 days	ICU stay >4 days	p	Hospital stay ≤37 days	Hospital stay >37 days	p
	(n = 23)	(n = 27)		(n = 37)	(n = 13)	
Age (yrs)	50 (45-58)	55 (48 - 61)	0.11	50 (45 - 58)	58 (51 - 63)	0.033
BMI (kg/m^2^)	23.8 (21.1-26.6)	26.4 (25.0 - 28.6)	0.04	25.4 (22.7 - 28.5)	25.5 (24.2 - 26.2)	0.72
MELD	14 (86-27)	26 (18 - 32)	0.007	18 (10 - 26)	28 (26 - 34)	<0.001
ICG-PDR (%/min)^1^	23.4 (18.7-26.7)	16.6 (8.8 - 22.8)	0.001	22.8 (15.5 - 25.3)	17.6 (10.1 - 20.8)	0.08
Bilirubin (μmol/l)^2^	94 (22-149)	133 (61 - 214)	0.03	88 (37 - 156)	162 (116 - 214)	0.008
Factor V (%)^3^	71 (41-92)	45 (27 - 66)	0.03	53 (30 - 73)	66 (49 - 83)	0.35

Data expressed as median (25^th^ - 75^th^ Percentile). Footnotes: ^1^) determined within 6 hours after ICU admission, ^2^) peak values within 7 days, ^3^) after 24 hours.

**Figure 1:**
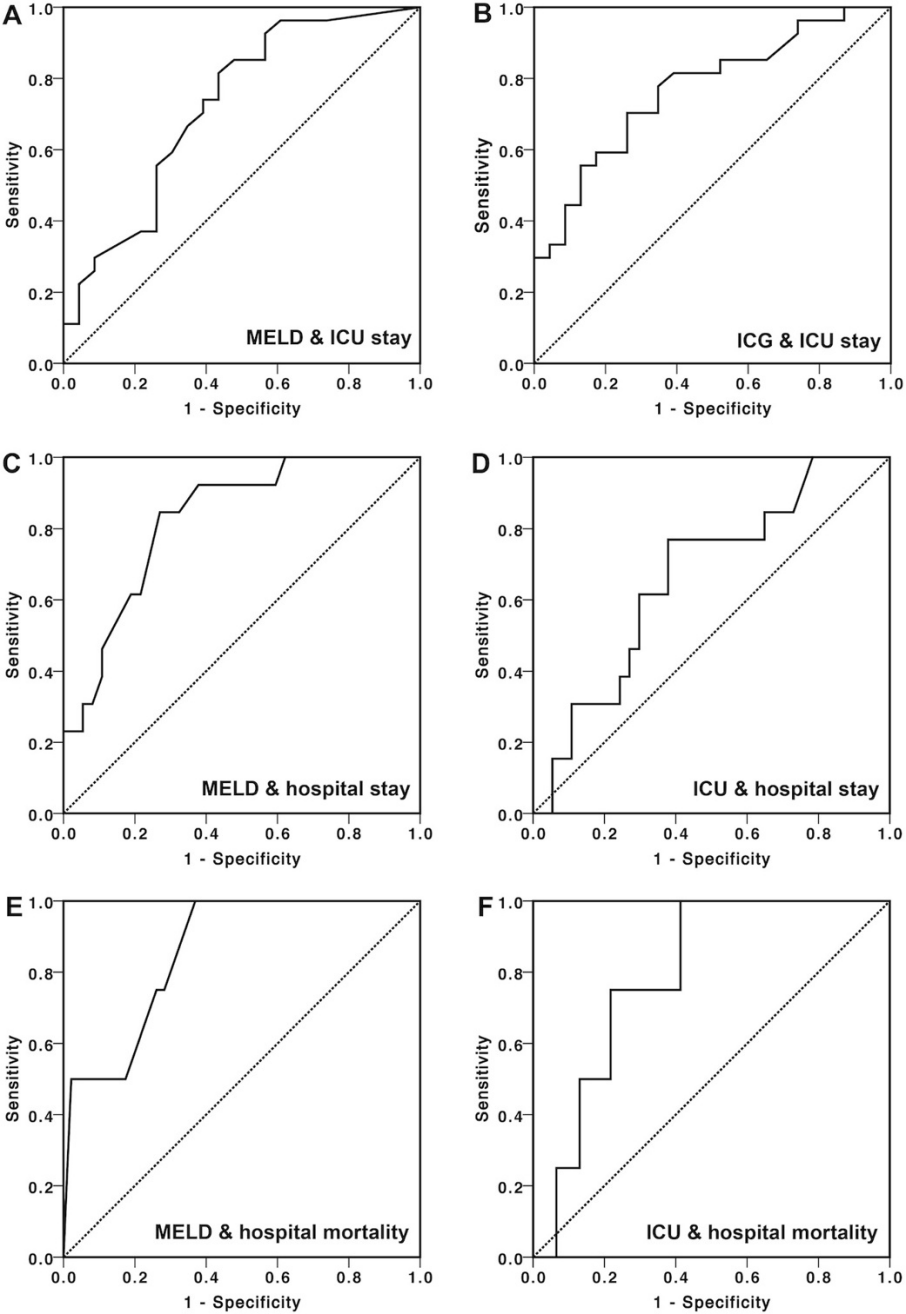
**ROC curve analysis of parameters with different clinical outcomes. (A)** Area under the curve (AUC) for MELD and ICU stay (≤4 *vs.* >4), **(B)** ICG and ICU stay, **(C)** MELD and hospital stay (≤37 *vs.* >37), **(D)** ICG and hospital stay, **(E)** MELD and hospital mortality, and **(F)** ICG and hospital mortality.

A ROC analysis was performed, which is presented in Table 4 and Figure 1. According to the determined cut off value, a MELD >25 was significant for prolonged ICU stay over four days (OR 4.12, 95% confidence interval (95% CI) 1.2 - 13.8, p = 0.024) and a prolonged hospital stay over 37 days (OR 13.0, 95% CI 2.5 - 68.6, p = 0.001). ICG-PDR < 20%/min was significant for prolonged ICU stay (OR 3.54, 95% CI 1.1-11.8, p = 0.047) and prolonged hospital stay (OR 4.67, 95% CI 1.20-18.34, p = 0.027) respectively. Peak bilirubin >100 μmol/l within the first postoperative week was significant for a prolonged hospital stay (OR = 0.063, 95% CI 0.007-0.54, p = 0.01) but not for prolonged ICU stay (OR = 0.39, 95% CI 0.12-1.21, p = 0.10). Factor V measurement 24 hours postoperatively failed to achieve significance for both length of stay in the ICU (OR = 0.35, 95% CI 0.11-1.13, p = 0.09) and length of hospital stay (OR = 2.13; 95% CI 0.56-8.16, p = 0.34). Four patients (8%) died during hospitalization, characterized by significantly higher MELD scores (p = 0.032) and significantly decreased ICG-PDR (p = 0.026) compared to survivors. Peak bilirubin (p = 0.12) and factor V (p = 0.31) were not significantly different between survivors and non-survivors.

**Table 4:** **Receiver operating characteristic analysis of MELD, ICG-PDR, bilirubin and factor V to predict the length of stay in the ICU and in the hospital as well as hospital mortality**

	Cut-off value	AUC	Sensitivity (%)	Specificity (%)	p
**ICU stay**					
MELD	25	0.67	63	74	0.05
ICG-PDR (%/min)^1^	20	0.77	70	74	0.001
Bilirubin (μmol/l)^2^	110	0.68	67	65	0.03
Factor V (%)^3^	40	0.75	67	74	0.002
**Hospital stay**					
MELD	23	0.81	100	54	0.001
ICG-PDR (%/min)^1^	20	0.67	77	62	0.08
Bilirubin (μmol/l)^2^	110	0.75	92	62	0.01
Factor V (%)^3^	50	0.52	77	40	0.80
**Hospital mortality**					
MELD	25	0.85	100	59	0.02
ICG-PDR (%/min)^1^	20	0.79	100	59	0.05
Bilirubin (μmol/l)^2^	110	0.68	100	54	0.25
Factor V (%)^3^	40	0.72	100	44	0.16

*Abbreviations:* AUC, area under the curve. Footnotes: ^1^) determined within 6 hours after ICU admission, ^2^) peak values within 7 days, ^3^) after 24 hours.

Based on these findings, a combination of MELD and ICG-PDR was tested. A positive MELD/ICG-PDR combination was defined as a high MELD score (MELD score > 25) and a decreased ICG-PDR (ICG-PDR < 20%/min), while all other possible combinations were defined to be a negative combination (Table 5).

**Table 5:** **MELD / ICG-PDR combination for prediction of length of stay in the ICU, length of hospital stay and hospital mortality**

	Positive combination	Negative combination	p
ICU stay, median (IQR)	9 (5-43)	4 (3-6)	0.004
Hospital stay, median (IQR)	42 (21-74)	22 (15-28)	< 0.001
Hospital mortality, n (%)	4 (40%)	0 (0% )	0.003

Positive combination: MELD >25 and ICG-PDR <20%/min. Negative combination: all other combinations. *Abbreviations:* IQR, interquartile range, SEM, standard error of mean.

A positive MELD and ICG-PDR combination predicted a twice as long ICU length of stay of median 9 days (p = 0.004) and a twice as long hospital stay of median 42 days (p < 0.001) compared to all other possible combinations. Hospital Mortality in case of positive combination was 40%, while it was 0% in case of negative combination (p = 0.003).

A multivariate analysis of ICU length stay selected the MELD/ICG combination as the most prediciting factor, while OR could not be calculated because all cases were discriminated. BMI was significant in this analysis with p = 0.007 (OR 9.61, 95% CI 1.88-26.5). Gender and age were not significant in this analysis (p = 0.16 and p = 0.11 respectively). Concerning Hospital length of stay, the MELD/ICG combination was significant with p = 0.006 (OR 64.17, 95% CI 3.3-1253) and age was significant with p = 0.045 (OR 22.63, 95% CI 1.08-415.2). Gender and BMI were not significant in this analysis (p = 0.61 and p = 0.60 respectively).

## Discussion

The major new findings of this study - undertaken to determine whether an easy-to-use combination of a singular ICG-PDR measurement early after transplantation and MELD correctly predicts postoperative ICU stay, hospital stay and hospital mortality - are that the positive combination of a high MELD score and a low ICG-PDR predicts a significantly longer stay in the ICU (9 days vs. 4 days) and a significantly longer hospital stay (42 days vs. 22 days) as well as a significantly higher hospital mortality (40% vs. 0%) compared to liver transplant recipients with a negative score.

The main limitations of our study are its small sample size and the post-hoc design, but nevertheless the results are promising and should be validated in another prospective collected dataset of liver transplant patients.

Our easy-to-use assessment combines the MELD score, for which it has been proven that high scores are associated with a prolonged postoperative course [4, 19] with an early postoperative ICG-PDR measurement of global liver function.

Low ICG-PDR values in the early postoperative phase of liver transplant recipients predict complications during the early postoperative period [23, 24] while normal values have been shown to predict an uncomplicated postoperative course [37]. Outcome prediction by ICG-PDR values has yielded conflicting results [24, 37, 38].

Measurement of ICG-PDR is an accurate test for evaluating liver function, but, as shown by Levesque’s results [23], its limitation is the lack to identify the underlying cause of a dysfunction. ICG-PDR is a test for global hepato-splanchnic blood flow and biliary excretion. Therefore, changes in ICG-PDR may be due to local disturbances in hepatic blood flow or systemic disturbances. It has been proved that absence of flow in the hepatic artery and primary graft dysfunction or non-function leads to diminished ICG-PDR [39, 40]. Moreover, it has been shown that ICG-PDR in patients with septic shock is reduced due to hepatocellular dysfunction [22]. For sepsis, it has been shown that recovery and survival is related to the course of repeated ICG-PDR measurements, where continuously low ICG-PDR over time is associated high mortality [22, 23].

Clinical benefit of our findings is risk stratification: Due to early assessment in the postoperative course, the combination of MELD scores and early postoperative ICG-PDR measurement might be valuable to identify risk patients in term of prolonged postoperative course and increased mortality and to prompt appropriate intensive care actions without time delay. This includes reassurement of hepatic blood flow by Doppler ultrasound, angiographic computed tomography or arteriography as well as calculated volume management and early goal-directed therapy in case of sepsis [41]. The negative combination of MELD scores and ICG-PDR seems to identify recipients with short postoperative course and low mortality. This negative predictive value is in agreement with the ICG-PDR data reported by Schneider [37] and may be useful as a criterion for transferring patients from the ICU to a peripheral ward and allows optimal utilisation of the ICU resources.

While the combination of the MELD score and a singular ICG-PDR measurement in the early postoperative phase and BMI were identified as predictors of ICU length of stay, the combination of MELD score and ICG-PDR and age were identified as predictors for hospital length of stay. Therefore, the MELD and ICG-PDR combination seems to be the best predictor and risk stratificator in liver transplanted recipients admitted to the ICU in terms of ICU length of stay, hospital length of stay and hospital mortality.

## Conclusion

In conclusion, the combination of the preoperative MELD score and a singular ICG-PDR measurement in the early postoperative phase (within six hours) is an interesting and easy to assess tool that should be addressed in a larger cohort of patients to evaluate its usefulness in terms of risk stratification and outcome prediction.

## Abbreviations

BMI: Body mass index
HBV: Hepatitis B virus
HCC: Hepatocellular carcinoma
HCV: Hepatitis C virus
HRS: Hepato-renal-syndrome
ICU: Intensive care unit
ICG: Indocyanine green
ICG-PDR: Indocyanine green plasma disappearance rate
INR: International normalized ratio
MELD: Model of end stage liver disease
RRT: Renal replacement therapy
SAPS II: Simplified acute physiology score II
SOFT score: Survival Outcomes Following Liver Transplant score
TPL: Transplantation.
